# Comparison of the Antibacterial Efficacy of Alexidine and Chlorhexidine Against Enterococcus Faecalis: An in Vitro Study

**DOI:** 10.7759/cureus.1805

**Published:** 2017-10-26

**Authors:** Preethi Varadan, Arathi Ganesh, Rudra Konindala, Venkateshbabu Nagendrababu, Rupa Ashok, Kandaswamy Deivanayagam

**Affiliations:** 1 Department of Conservative Dentistry and Endodontics, Faculty of Dental Sciences, Sri Ramachandra University, Porur, Chennai, India; 2 Division of Clinical Dentistry, School of Dentistry, International Medical University, Kuala Lumpur, Malaysia

**Keywords:** alexidine, antimicrobial efficacy, chlorhexidine, enterococcus faecalis

## Abstract

Introduction

Root canal irrigants play an important role in reducing intracanal microorganisms, which in turn helps in achieving a successful outcome for the root canal treatment.

Objective

To compare the antibacterial efficacy of alexidine and chlorhexidine against Enterococcus faecalis.

Methods

A total of 50 extracted single-rooted teeth were randomly divided into five groups after being infected with Enterococcus faecalis. The groups were based on irrigants used: Group I - 0.4% alexidine; Group II - 1% alexidine; Group III - 1.5% percent alexidine; Group IV - 2% alexidine; Group V - 2% chlorhexidine. Following irrigation, colony-forming units were determined from the dentinal shavings collected at 400 µm depth.

Results

Use of 2% alexidine reduced the bacteria effectively when compared to 0.4%, 1%, and 1.5% alexidine. A statistically significant difference was not observed between 2% alexidine and 2% chlorhexidine.

Discussion

Alexidine, due to its higher virulence factors for bacteria and better bacterial penetrability at 400 µm depth of dentin showed better eradication of Enterococcus faecalis in comparison to chlorhexidine.

Conclusion

The use of 2% alexidine against Enterococcus faecalis at 400 µm depth of dentin has efficacy comparable to chlorhexidine. Hence, alexidine can be used as an alternative irrigant for chlorhexidine during endodontic procedures.

## Introduction

The main causative agent of several pulpal and periapical diseases has been reported to be bacteria [1]. Microbial infection, incessant in the root canal system and/or periradicular area [2],  is one of the predominant reasons for the failure of endodontic treatment. It is possible to reduce the bacterial population through cleaning and shaping; however, complete elimination is not possible [3-4]. The persistent endodontic infection provoked by bacteria within the dentinal tubule is responsible for the resurgence of the apical periodontitis [5]. Enterococcus faecalis is found as a major remnant flora in endodontically treated teeth [6-7]. Even in the most intolerant of conditions such as well instrumented and obturated root canals, the Enterococcus faecalis species thrive well with very little available nutrients [8-9]. Persistent intraradicular infections showed higher prevalence of Enterococcus faecalis when compared to untreated chronic periapical periodontitis [10].

Root canal irrigants play a vital role in reducing the microorganisms inside the root canal system [11]. Chlorhexidine is the most commonly employed antimicrobial root canal irrigant showing substantivity [11]. Silveira, et al. elicited that alexidine, a biguanide disinfectant, had a better antibacterial effect against Enterococcus faecalis [12-13]. Barrios, et al. showed that alexidine has also shown antimicrobial substantivity similar to chlorhexidine against Enterococcus faecalis [14]. A literature search revealed that the interaction of sodium hypochlorite with alexidine did not produce any precipitate. Another important requisite for any root canal irrigant is for it to maintain the same antimicrobial efficacy, even after it penetrates deeper into the dentinal tubules. The current study aimed to compare the antibacterial efficacy of alexidine with chlorhexidine against Enterococcus faecalis at 400 µm depth of root canal dentin.

## Materials and methods

The extracted single-rooted premolars were cleaned using scalers and 3% sodium hypochlorite (NaOCl) (Prime Dental, Thane, India), following which sectioning of the teeth at the level of the cemento-enamel junction was performed using a diamond disc. Then, the lengths of the teeth were standardised to 12 mm. The specimens were then irrigated with 3% NaOCl for five minutes, and the smear layer removal was carried out using 17% ethylene diamine tetra acetic acid (EDTA) (Ammdent, Mohali, Punjab, India) for one minute. The existing traces of the remaining chemicals that were used were removed from the teeth specimens using distilled water, and they were subsequently sterilized in an autoclave.

The Enterococcus faecalis (MTCC 3159) (Microbiologics, MN) bacterial strain was grown in a brain heart infusion broth (BHIB) (HiMedia Laboratories, India) for 24 hours at 37°C. Serial dilution of alexidine (alexidine dihydrochloride, Santa Cruz Biotechnology, Heidelberg, Germany) was performed as follows: 100 μl of each dilution was added to 100 μl of the Mueller‑Hinton broth. To this broth, 5 μl of bacterial suspension was added and the mixture was incubated at 37°C for 24 hours. After 24 hours, the tubes were visually checked for bacterial growth. All the specimens were immersed in the Enterococcus faecalis inoculation. To achieve biofilm formation, contamination of all specimens was continued for a period of 21 days. The samples were randomly divided into five groups: Group I - 0.4% alexidine; Group II – 1% alexidine; Group III – 2% alexidine; Group IV – 3% alexidine; Group V – 2% chlorhexidine (Ammdent, Mohali, Punjab, India). Alexidine dilutions were prepared in the concentrations of 0.4%, 1%, 1.5%, and 2% by weighing the amount of powder required and mixing with 100 ml of distilled water. After irrigation, the dentinal debris were harvested at the depth of 400 ųm by using a Gates Glidden drill size five. Then, 1 ml of sterile BHIB was used to collect the dentin debris, which was further incubated at 37˚C for 24 hours in an anaerobic environment. Further, serial dilution to 100 µL of broth in 100 µL of normal saline of the content of each micro-centrifuge was carried out five times. Then, brain heart infusion agar plates were used to plate 5 µL of the diluted sample, and it was incubated for 24 hours before the colonies were counted [15-16]. Data analysis was performed with oneway analysis of variance (ANOVA) and post-hoc Tukey honest significant difference (HSD) method using SPSS version 16.0 (IBM, Armonk, NY). A p value < 0.05 was considered to be statistically significant.

## Results

The quantitative data of the remaining bacterial count in each group is presented in Table 1. Group IV showed the least number of colony-forming units of Enterococcus faecalis when compared to other concentrations of alexidine. However, 2% alexidine and 2% chlorhexidine did not show any statistically significant difference in the number of colony-forming units.

**Table 1:** Statistical analysis using oneway ANOVA and post-hoc Tukey HSD method * p value < 0.001 ** p = 0.292 *** p = 0.995 Group I: 0.4% alexidine, Group II: 1% alexidine, Group III: 1.5% alexidine, Group IV: 2% alexidine, Group V: 2% chlorhexidine cfu: colony-forming unit

Groups	Log CFU/Slide		p-value	Comparison Among the Groups
	Mean	Standard Deviation		
Group I	8.077	0.926	0.000*	Group I vs Group II**
Group II	7.198	1.377		Group I vs III, IV, V*
Group III	4.880	1.007		Group II vs III, IV, V*
Group IV	2.802	0.767		Group III vs IV, V*
Group V	2.627	0.753		Group IV vs V***

## Discussion

The current study aimed to check the antibacterial property of alexidine at a depth of 400 µm into the dentin. In the present study, the root canal inner diameter was standardized to a diameter equivalent to a Gates Glidden drill size three. The Enterococcus faecalis biofilm was formed and disinfected with the experimental irrigants. A Gates Glidden drill size four was used to remove the dentinal debris which corresponds to 200 µm, but this dentinal debris was not used for analysis in the present study. Later, Glidden drill size five was used to remove the dentinal debris, which corresponds to 400 µm, and this debris was used for the analysis. The above-mentioned procedure is the proven and standardized methodology for checking the antibacterial efficacy of disinfectants at two different depths [17-18].

From the results obtained in the present study, it was concluded that 2% alexidine and 2% chlorhexidine did not show any statistically significant difference in Enterococcus faecalis eradication. Group III demonstrated the best antibacterial activity against Enterococcus faecalis. The structural difference between alexidine and chlorhexidine is the presence of two hydrophobic ethyl hexyl end groups. Alexidine's chemical structure, hydrophobic penetration into membrane lipids, and electrostatic attraction to the negatively charged cell membrane contributed to cell lysis, which in turn was responsible for its antibacterial nature [19-20]. Alexidine has the ability to alter the permeability of the bacterial cell membrane faster [21]. In the present study, no statistically significant difference was observed between alexidine and chlorhexidine. The results of the present study were concurrent with Kim, et al. [22]. Compared to chlorhexidine, alexidine has a higher affinity for the virulence factors of bacteria [23]. A study by Barrios, et al. concluded that alexidine had longer antimicrobial substantivity against Enterococcus faecalis when compared to chlorhexidine [14]. While Bui, et al. in their study showed that a combination of NaOCl and chlorhexidine resulted in the formation of a precipitate which occluded dentinal tubules [24], Kim, et al. showed that no kind of precipitate was observed from the alexidine and NaOCl combination [13]. 

Studies showed that the cytotoxicity effect of chlorhexidine was dose-dependent [25-27]. Tu, et al. showed that a higher concentration of chlorhexidine inhibited stem cell proliferation significantly in comparison to lower concentrations [28]. Considering this issue, the authors studied the antimicrobial efficacy of various concentrations of alexidine. The results of the current study showed that 2% alexidine had better antibacterial property against Enterococcus faecalis in comparison to the other groups. It was beyond the scope of the current experiment to study the effect of various concentrations on stem cell viability. More studies in this regard should be performed to confirm the cytotoxicity of alexidine at various concentrations.

## Conclusions

Alexidine showed better antimicrobial property at 2% when compared to other concentrations of alexidine. No difference was observed between 2% alexidine and 2% chlorhexidine at 400 µm. Considering the advantages of alexidine (antimicrobial property, longer antimicrobial substantivity, absence of precipitate formation), it might be used as an alternative irrigant to chlorhexidine.
